# Active biopackaging produced from by‐products and waste from food and marine industries

**DOI:** 10.1002/2211-5463.13121

**Published:** 2021-04-01

**Authors:** Frédéric Debeaufort

**Affiliations:** ^1^ Department of BioEngineering IUT‐Dijon‐Auxerre University of Burgundy Dijon Cedex France; ^2^ Joint Unit A02.102 PAM‐PAPC ‐ Physical Chemistry of Food and Wine Laboratory Univ. Bourgogne Franche‐Comté/AgroSupDijon Dijon France

**Keywords:** agro‐food by‐product valorization, antimicrobial and antioxidant, bioactive films, biopolymers, food packaging, sustainability

## Abstract

The agro‐food industry cannot today do without packaging to preserve and above all market its products. Plastic materials coming mainly from petrochemicals have taken a predominant place in the food packaging sector. They have become indispensable in many sectors, from fresh to frozen products, from meat and dairy products to fruit and vegetables or almost‐ready meals. Plastics are cheap, their lightness reduces transport costs, and their convenience is fundamental for out‐of‐home catering. However, plastics pose serious end‐of‐life issues. The development of materials that are more respectful of the consumer and the environment has become a major issue. In addition, the agro‐food industries generate significant quantities of waste or by‐products that are poorly or not at all recovered. However, these contain constituents that can be extracted or transformed to be compatible with packaging uses. Many molecules from waste materials are of particular interest for the development of active packaging such as biopolymers, bioactive agents, inorganic compounds, fibers, or nano‐ and micro‐objects. Providing bioactive functions such as antioxidants or antimicrobials can extend the shelf life of food while reducing the sophistication of plastic materials and thus improving their recycling. This article summarizes the main materials and constituents that can be recovered from waste and illustrates through several examples what could be the applications of such new, sustainable, and active packaging.

## Graphical abstract

The main trend for packaging materials deals with sustainability. Agri‐food and seafood industries generate high amounts of waste that could be valorized for packaging applications. Indeed, from cereals, milk, meat, or fish waste, we can produce, for instance, biopolymers that constitute the matrix of packaging materials, antimicrobial agents providing bioactive properties, and natural additives that could substitute chemicals used for plastic.

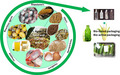

**Abbreviations**
HDPE: High‐density polyethylene
LDPE: low‐density polyethylene
PE: polyethylene
PET: polyethylene terephthalate
PHA: polyhydroxy alkanoate
PHB: polyhydroxy butyrate
PHBV: polyhydroxybutylvalerate
PLA: polylactic acid (polylactides)
PP: polypropylene

By 2050, we will need to feed 2.2 billion additional people, and with the increase in standards of living, this will mean an increase in food needs of more than 50%. This requires not only better use of agricultural land, but also and above all an optimization of the conservation of raw materials and finished food products. The latter requires in particular the use of adequate packaging capable of protecting the food, ensuring a longer shelf life, while at the same time meeting societal expectations, including the preservation of the environment and health.

Today, only plastic materials mainly derived from oil (mainly‐low and high‐density polyethylene, LDPE and HDPE) seem to be able to satisfy this necessary growth in agro‐food production. Indeed, the production of plastic polymers for packaging uses has been growing steadily for 40 years, from around 50 million tons in the early 1980s to more than 360 million tons in 2019 [1]. Thirty percent of all plastic produced in 2018 came from China, compared to 17% from Europe, where production has declined over the last 5 years. In 2018, 9.4 million tons of plastic postconsumer waste were collected in Europe for recycling, as consumers and the industry pay more attention to plastic disposal and end use [2]. But this still only covers a small percentage (< 20% of production), the rest being at best incinerated, at worst released into nature. The packaging waste generated per capita has increased overall during this period, rising to 32.7 kg per capita in 2017 in the EU. Fortunately, the volume of plastic packaging waste collected for recycling has noticeably increased, with waste sent to landfills decreasing by more than 50% to 3.3 million metric tons in 2018. However, collected and recycled plastic amount remains much less that the amount produced and used during the same period.

Indeed, according to the Ellen MacArthur Foundation [3], the oceans will contain the same mass of plastic waste as fish in 2050. However, it is not possible to fish for plastics the way we fish for fish, or even to recover the plastic ingested by the fish, but rather to reduce their accumulation in the seas. The proportion of plastic waste that ends up in the oceans from packaging is almost 50%; the rest are synthetic fibers from washing clothes (plastic microfibers) and from other industries. Only 10% of the waste comes from the fishing industry and marine transportation (including yachting), the remaining 90% comes from the land, after being transported by rivers [4]. Eighty percent of packaging is single use, and more than 75% of the packaging produced is destined for the food industry. Figure 1 illustrates the rate of plastic production, the rate of ocean pollution by macro and microplastics, as well as the rate of plastic degradation (based on an average of 450 years required for full degradation of a polyethylene material).

**Fig. 1:**
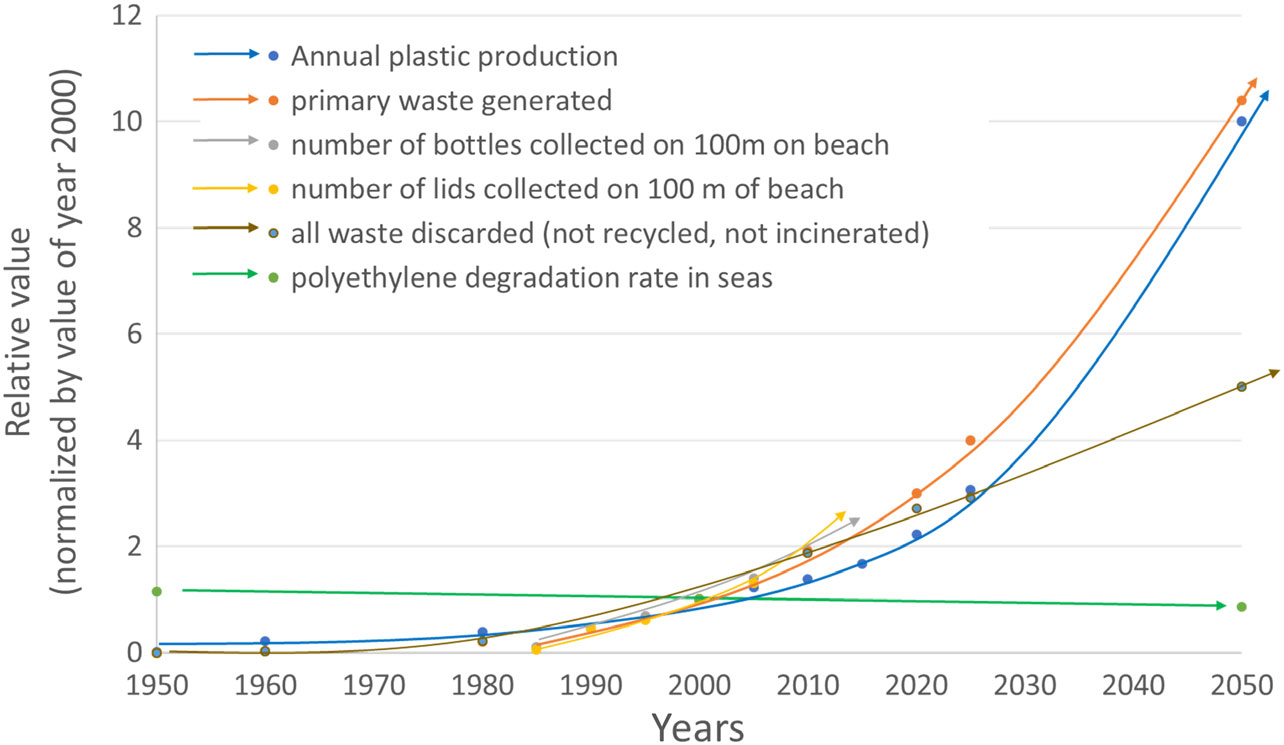
Trajectories of plastic production [5], primary waste generation by consumers and industries [6], number of bottles and lids collected from a region 100 m long on South African beaches [7], waste discarded (adapted from Waters *et al*. [8]), and of the theoretical degradation of polyolefins in oceans.

Packaging has therefore become a social issue, but it is also the reflection of a brand regarding a product. In order to regain consumer confidence in food products, packaging must be in line with the product. This is even truer for products resulting from sustainable development: the packaging must also be sustainable.

There are no good or bad raw materials, but are there right or wrong packaging? Indeed, all the materials used for the manufacture of packaging have more advantages than disadvantages, as well as interesting characteristics and recognized performances. It is the destination that the consumer sends them to after use that is problematic, not their intrinsic nature and properties. As packaging is reflective of our society, should we go back to square one with only protection functions or do we have still room for exciting design and new properties and functions?

To understand the likely scenarios, we would need to look at 10 drivers below which impact the future of packaging:

- Climate change impact and increase resource efficiency: need to reduce product and packaging waste—increase recycling—clean landscape, remove wasted space (filling economy), and optimize transport—develop new sustainable supply chains
- Instant communication of fake information misleading consumers: could make new certification easier to understand—verified product and packaging—trusted web site—trusted packaging—trusted product
- New technologies: developing interactive (active) and smart packaging to avoid product waste, counterfeiting and to support medical prescription, easier access to information. Three‐dimensional printing and robotics will impact on demand for packaging, removing wasted space in packaging and the supply chain, provide flexible machines.
- Next financial crisis may be close; two main leading streams: luxury and trendy packaging design for luxury goods and basic packaging for all commodities including food.
- Increase of Earth population: access to food and fresh water will be the main issue in several parts of the world. Avoidance of water and food wastage will be vital and this needs new production systems with efficient supply chains.
- Legislation for additional bans and to increase taxes—need simpler packaging, remove unnecessary packaging
- Security—need for transparency and increase food safety—use block chain data for products and packaging
- Protectionism—people prefer local *vs* global—individual and local production will impact short and direct distribution supply chains and thus packaging.
- People gratification—there is still a need for enhanced self‐experience, convenience, and ease.
- Social—adequate packaging regarding diet, religion, gender, etc.

Packaging can be easily recyclable and recycled (industrial) but the problem becomes more complicated at the end of life (after several cycles of recycling) because most recyclable packaging, apart from paper fibers, is not biodegradable and vice versa.

Two main directions are emerging for manufacturers to choose packaging that minimizes the impact on the environment and reduces waste:

- Reusable packaging (industrial or private).
- Biodegradable packaging for which it is necessary to define the mode of degradation:
  ○ Home composting or industrial composting [degradation by microorganisms in an aerobic environment (with air)]
  ○ Methanization: degradation by microorganisms in an anaerobic environment (without air) to produce gas.
  ○ Degradability in the marine environment (conditions and standards are still under development)

The origin of the packaging can also come from several sources: renewable or fossil. If the material is biosourced, certifications attesting to a sustainable origin (e.g., Forest Stewardship Council or Programme for the Endorsement of Forest Certification for paper) will also be required, as well as knowledge of the impact of the raw material on vital human needs (water–air–food).

The holy grail of sustainable packaging would be biodegradable, barrier, and recyclable packaging, of renewable origin while not using food resources. Indeed, the biodegradable packaging films on the market today, based on starch or polylactic acid (PLA), are made from cereals (mainly corn) that could be used for human food, given that 10% of humans suffer from malnutrition.

The use of waste or by‐products from the food processing industries for packaging applications is an avenue being studied by many research laboratories, which is attracting major interest from the food processing industries and, to a lesser extent, from industries producing packaging materials. The biorefinery concept is of key importance to answer these challenges, to produce commercial products suitable for the packaging industry from waste or by‐products from agro‐food sector.

## Components recovered from waste and by‐products of agro‐food and marine industries suitable for food packaging

Many compounds from waste recovery can be used for packaging materials. These include biopolymers, fibers, nanoparticles, bioactive compounds, and inorganic compounds. In general, biopolymers come to mind first and foremost as the basic material for packaging, since they form the matrix or network (continuous structure). The other constituents mentioned above are rather considered as additives (plasticizers, active agents, cross‐linking agents, etc.) or fillers (fibers) in the manufacture of packaging materials. Table 1 summarizes the compounds potentially recovered from waste, their function in packaging, and their origin.

**Table 1:** Potential constituents recovered from agro‐food industries for packaging applications.

Packaging parts	Properties for packaging applications	Nature	Origin	References
Polymers and biopolymers	Structural properties, continuous structure, and network Transfer and migration control	PHA, PHB, PHBV PLA Cellulose Chitin/Chitosan Starch Collagen/Gelatins Caseins Corn zein, etc.	Seafood and fish, milk and dairy, cereal, meat, sugar industries. Exhaust water from food industry	[9, 10, 11, 12, 13, 14, 15]
Fibers	Fillers, structural reinforcer, barrier properties, encapsulation matrix, moisture and fogging control	Cellulose Lignin Powders of fruits stones, pits, or shells Bran, husk	Cereal crops, Sugar cane (bagasse) Fruits	[16, 17, 18, 19]
Nanoparticles	Fillers, barrier and mechanical properties, active properties, encapsulation support	Cellulose nano whiskers, lignin Protein or polysaccharide nano‐ and micro‐objects or beads (gelatin, pectin, chitosan, etc.)	Cereal industry, crop productions Seafood, fish, and meat industries Paper industry	[19, 20, 21]
Bioactive compounds	Antimicrobial, antifungal, antioxidant, antibrowning, antitumoral, etc., properties	Phenolic compounds, lignin Essential oils Enzymes, peptides, protein hydrolysates	Food, agro, seafood and fish, meat, and milk industries Paper and wood industries	[9, 12, 14, 22, 23, 24, 25]
Additives	Plasticizers Antistatic, wetting agent	Polyols, fatty esters, emulsifiers	Oil and soap industry Seafood and fish industries	[25, 26, 27]
Inorganic compounds	Light or oxygen barrier, Inert fillers	Calcium, sodium, potassium carbonates, calcite, zeolites	Husks from cereals Fish scales and bones Mammalian bones	[25, 26, 28]

### Biopolymers

Biopolymers obtained from biomass from the food processing industry are the key element for the constitution of packaging. Biopolymers provide a solution to waste problems but require the establishment of a waste management system suitable for this type of product. Thus, the organization of a collection and processing chain is essential to ensure optimal recovery of these biopolymers for use in biodegradable packaging [29].

Based on their origin (vegetal or animal and the waste type) and the production method, biopolymers can be classified into four groups [30, 31]:

1. Biopolymers obtained directly by concentration from biomass (polysaccharides, e.g., starch, cellulose, chitin and chitosan, pectin, etc., or proteins such as collagen and gelatin, casein, gluten, etc.). This group is the most promising for packaging but limited by their cost of extraction and water sensitivity. Several starch‐based films are already on the market.
2. Biopolymers obtained by the usual chemical synthesis from monomers. Carbohydrate fermentation is the most common way to obtain such a monomer. The most developed and commercialized biopolymer is PLA (polylactide).
3. Biopolymers produced by the activity of microorganisms. The main representatives of this group of polymers of biological origin are mainly polyesters such as polyhydroxyalkanoates PHAs (polyhydroxybutyrate PHB, polyhydroxybutylvalerate PHBV); however, most materials based on bacterial cellulose are currently under development and their cost remains a limitation for industrial production.
4. Biopolymers produced by chemical synthesis from biological monomer products derived from biomass hydrolysis, such as polybutylene succinate, bio‐based terephthalic acid, bio‐based PP and PE, etc. However, this last group is currently only slightly derived from waste recovery and they are not all biodegradable.

Biopolymers have interesting properties for packaging applications. Apart from their primary function of mechanical protection of products thanks to their ability to form continuous and resistant networks, biopolymers offer other functions linked to their intrinsic properties [32]. For example, water vapor permeability (useful for packing fresh products such as fruit and vegetables), permselectivity (ratio of gas permeabilities, which is very different to that of synthetic polymers) [33], transparency and gloss, shape memory (high folding capacity), limitation of surface condensation thanks to their water absorption capacity, sealability at relatively low temperatures [34], barrier properties to odors and aromas [35], barrier properties to fats and oils [36], low coefficient of friction and antistatic (which facilitates printing [37]), and an ability for encapsulation [35].

Biopolymers are then used mainly as a structural matrix, but could also be used for their interesting interactions with active compounds such as antimicrobial or antioxidant agents, natural pigments, flavors, and neutraceutics [38]. Therefore, their properties for encapsulation, entrapment, and consequently controlled released are highly sought after by the food packaging industry for active bio‐based packaging materials [39, 40].

### Fibers

Offered in the form of powders resulting from the extraction or grinding of mainly plant matter, natural and sustainable fibers provide solutions for the development of biodegradable packaging. They are used as a reinforcing material for bio‐based composites, as vegetal fillers for biopolymers and resins, or as texturizing agents to create various haptics and surface structures. Initially used as a bulking agent (fillers) to reduce the amount of polymer, the addition of fiber is now being considered for other functions. Indeed, the incorporation of fibers in biopolymers either improves their mechanical and/or barrier performance or provides a balance between various functional properties. The incorporation of fibers on a micro‐ or nanoscale in polymers or biopolymers is a real challenge for the future of packaging materials, particularly for biodegradable packaging [41]. At the nanometric scale, fibers of natural origin have been of interest for use in synthetic polymers but also in biopolymers providing sensory properties such as soft touch or clarity. Fibers incorporated in biopolymers have other very interesting functionalities, for example, for the incorporation or encapsulation of active molecules such as antimicrobials or flavors [35]. These composite materials made up of a biopolymer and fibers have release (diffusion and partition) properties that are compatible for developing active packaging. The fibers most widely used to make composite materials are essentially derived from cellulosic materials (wood, straw, bran, etc.) [42]. However, there are also composites comprising chitosan, lignin, pectin, or zein fibers. They have been tested in various biopolymers such as PLA, PHBV, etc. [43, 44]. Indeed, lignin fibers were also recently used to provide either toughness or antioxidant properties to PLA films [45].

Currently on the market for packaging purposes are new fibers obtained from by‐products of the fruit or oil industry, such as powders from walnut, pistachio or argan shells, apricot, peach or avocado stones, or olive pits [46]. For instance, gelatin films containing barley bran fibers display a significant capacity to reduce proliferation of *Escherichia coli* O157:H7 and *Listeria monocytogenes* inoculated on packaged salmon [47].

### Nanoparticles

Nanomaterials are defined as materials with an external dimension at the nanoscale (nano‐objects) or with an internal structure or surface structure at the nanoscale (nanostructured materials). They are classified into several categories [19, 48]:

1. Nanoparticles are nano‐objects whose length, diameter, and/or width are at the nanoscale and are not or only slightly different from each other.
2. Nanofibers are nano‐objects with two external dimensions at the nanoscale and the third, significantly larger dimension (several nanometer or micrometers).
3. Nanoplates have only one external dimension at the nanoscale (thickness) and the other two external dimensions significantly larger, even at the micrometer scale.

Other nano‐objects are nanorods, nanotubes, and nanowires, which are special cases with specific properties such as high rigidity, hollow bodies, and (semi‐) conductive properties. Nanoribbons are a special case of plates where one plane dimension is significantly larger than the other.

The addition of nanomaterials, such as nanoclays or titanium dioxide, can improve the properties of a material, for example making it lighter and stronger. In addition, nanomaterials can prevent gases and light from entering the packaging and causing degradation, and they can also have antimicrobial properties, thereby helping to keep food fresh for long periods of storage and free from harmful bacteria. The vast majority of nanomaterials used for packaging are inorganic (nanoclays, titanium dioxide, etc.), but a few come from the recovery of waste from agro‐industries. However, there are nanomaterials of biological origin, which are the subject of increasing attention from scientists.

These include nanofibrous cellulose, cellulose nanocrystals, or nanowhiskers, for instance. These materials, extracted directly from natural fibers, can improve resistance and gas barrier, but are still very sensitive to humidity. Their application in films and coatings is described as nanocomposite systems. Although they are still in their infancy, a great deal of research has been carried out on these bio‐materials [49, 50].

Biopolymers have been widely used for the synthesis of various nanoparticles in the pharmaceutical sector and are increasingly transferred to the food and packaging sectors. Biopolymers such as chitosan, starch, cellulose, gelatin, polyvinyl acetate, polyvinyl pyrrolidone, etc., can thus replace various reagents and synthetic, even toxic, polymers in the synthesis of various nanoparticles [51]. Indeed, lignin nanoparticles incorporated into chitosan/polyvinyl alcohol hydrogel for packaging coating have demonstrated antioxidant properties [52].

Several natural nanomaterials based on proteins or polysaccharides considered as by‐products of the food industry have been included in biopolymers (PLA, PHAs, chitosan, starches, etc.) to make nanocomposites [48]. For example, to improve the properties of PLA, the incorporation of chitin nanocrystals has been considered. The modification of the chitin particles allows better compatibility between the nanoparticles and PLA which results in a positive effect on the mechanical, thermal, and optical properties of the reinforced PLA films [53].

Starch has been studied for decades as a material of choice for food packaging applications, including in the form of nanomaterials, for instance as encapsulation matrices. There are several associated advantages known: abundance, biocompatibility, nontoxicity, low cost, biodegradability, availability, and stability explain the interest in this biopolymer for packaging. Native starch granules can be subjected to hydrolysis allowing the separation of crystalline lamellae. The crystalline starch particles have a platelet morphology with thicknesses of 6–8 nm, which when incorporated in pullulan‐based films, improves their mechanical resistance. In addition, the positive surface charge contributes to antimicrobial activity [54].

Chitosan, a heteropolysaccharide, is known for its biocompatibility, biodegradability, and polycationic nature which gives it a broad spectrum of antimicrobial and antifungal activities [55, 56]. Chitosan nanoparticles are formed by ionic gelation, where the positively charged amino groups of chitosan interact electrostatically with the polyanions used as cross‐linking agents [55, 56]. The incorporation of chitosan nanoparticles embedded in a polyvinyl alcohol‐based film can extend the shelf life of tomatoes by controlling the development of *E. coli*, *S. aureus*, and *Bacillus subtilis* [57].

Bags made of chitosan particles in polyethylene (LDPE) have been shown to be effective in inhibiting total aerobic mesophilic bacteria, coliforms, molds, and yeasts in chicken drumsticks while preserving sensory qualities [58]. Rieger *et al*. [59] encapsulated cinnamaldehyde as an antimicrobial agent in chitosan nanoparticles to limit the growth of several microbial strains including *E. coli, S*. *aureus, P. aeruginosa, Aspergillus niger*, and *Candida albicans*.

### Natural additives and bioactive agents

Plants are the most abundant bioresources, providing valuable compounds that can be used as additives in polymeric materials, such as fibers discussed above, as well as plant extracts containing bioactive phenolic and flavonoid compounds that have long been used in health products, as well as in the pharmaceutical, cosmetics, and nutraceutical industries. The incorporation of additives into polymer materials improves their properties to make them suitable for a wide range of applications, including packaging. Efforts are being made to incorporate various natural biosourced and biodegradable additives with a low environmental footprint, such as by‐products, biomass, plant extracts, etc., into raw polymers [60].

The demand for the use of natural additives in polymer formulations has led in recent years to a marked increase in the number of studies based on natural plant extracts, essential oils, or agricultural waste and their original compounds (including organic acids, phenolic acids, tannins, proanthocyanidins, and flavonoids) for food packaging applications [61]. Peptides, protein isolates, and hydrolysates such as those extracted from fish and seafoods have natural antioxidants and/or antimicrobial properties, and were envisaged by Hajji *et al*. [unpublished data] as useful for packaging applications or for wound dressings.

Their action is essential to prevent food oxidation, the development of bad taste or off‐flavors, nutritional losses, food‐borne bacterial spoilage, and organoleptic deterioration by the proliferation of microorganisms. It is these properties that are targeted in the development of natural active bio‐based packaging incorporating these bioactive molecules.

Natural plasticizers, such as polyols and fatty esters, can also be obtained from the vegetable oil extraction industry and are suitable for packaging applications as a substitute for chemical plasticizers. Indeed, from the by‐products (leaves, pomaces, pruning, stone, kernel) of olive oil processing, many compounds suitable for packaging additive substitutes can be used, such as polyols, phenolic extracts, fatty acids and esters, tannins, pectic polymers, hemicelluloses simple phenols (tyrosol, hydroxytyrosol), lignans (pinoresinol, acetoxypinoresinol, syringaresinol), secoiridoids, flavonoids (flavones, favonols such as rutin, kaempferol, quercetin, or flavan‐3‐ols: catechin), cinnamic acid derivatives (verbascoside, isoverbascoside) and phenolic acids (caffeic acid, cinnamic acid, syringic acid, chlorogenic acid, ellagic acid, vanillic acid, coumaric acid, ferulic acid), many of which have been tested in active packaging systems [27, 40].

### Inorganic compounds (minerals and oligoelements)

Very few inorganic compounds arising from food industry residues have been considered for food packaging applications. However, food industry waste is also a potential source of inorganic compounds, such as hydroxyapatite and calcium carbonate, which can be recovered from mammalian bones, fish bones, and scales [25]. Piricillo *et al*. [28] have shown that fish bone powder (hydroxyapatite) extracted from the waste of various fish (bones, scales) is an excellent sunlight screen and could be considered for applications as food packaging that serves as a barrier to gas and to UV light while maintaining the transparency of the material.

The shell of gallinaceous eggs is essentially composed of 95% calcium carbonate (calcite), magnesium carbonate, and sodium carbonate. Although a large proportion is reincorporated into poultry feed, these inorganic elements can be considered for incorporation into polymers and biopolymers for packaging [62]. These carbonates can not only be considered as fillers, but also have barrier properties to oxygen and to light and UVs, as shown by Aframehr *et al*. [63], when incorporated into PLA. The addition of eggshell nanoparticles to a bioplastic (PLA, PHBV, etc.) further increases the mechanical strength and flexibility of the composite compared to the biopolymer alone, making it potentially interesting for use in the packaging industry. This new packaging also retains its biodegradation properties, while gaining functional properties such as gas and UV impermeability and mechanical strengthening [64]. Also, husks from rice or sugar cane contain inorganic elements such as cements, concretes, zeolites, or carbonates which can be recovered after green chemical treatments such as hydrolysis, and these are suitable for packaging as nanoparticles or fillers [26].

## Example of some active packaging based on compounds obtained from fish and seafood waste and by‐products

The problem of waste from fishery and seafood industries has increased in the last decade, becoming a global concern [65]. Excluding aquatic plants, global production of fish, crustaceans, mollusks, and other aquatic animals reached almost 175 million tons in 2017, among which 25% are wasted [66]. ‘Fish waste’ includes fish species or by‐catch products with no or low commercial value, undersized or damaged fish, and species of commercial value but not caught in sufficient amounts to warrant sale. Fish and seafood industry waste is related to the waste and by‐products resulting from the processing and storage of fish and sea‐based products.

As a result of filleting, salting, smoking, and canning operations, the seafood processing industries manufacture various processed products such as fillets, steaks, fish nuggets, etc. As a consequence of this activity, they generate a large number of by‐products and waste [67]. The latter represents more than 60% of the total mass of fish and includes skin, bones, viscera, head, scales, etc. [68, 69]. It has been estimated that more than 66% of fish, crustacean, and cephalopod tissues among the 120 million tons processed in 2017 including fins, heads, skin, and viscera are discarded as they are considered ‘waste’ whereas they contain high amount of collagen that could be converted into gelatin [70]. The processing of only crustaceans and mollusks in the European Union generates significant amounts of solid waste, estimated at 0.5 million tons/year, from which a huge quantity of chitin could be extracted and thus chitosan obtained [67]. Composting, incineration, and landfilling have been considered the easiest and cheapest solutions to dispose of these huge amounts of waste, assuming that the by‐products are waste to be managed rather than recovered [67]. Nevertheless, these methods threaten the environment, particularly incineration, since it releases carbon monoxide and dioxide into the atmosphere, or discharges into the sea, which unbalance ecosystems. Thus, the recovery of these by‐products, given their richness in high‐quality proteins, lipids, minerals, etc., may be a better alternative to the traditional options of dumping at sea or in waste disposal sites. Consequently, various studies have been carried out over the last few decades to identify new biotechnological processes for the valorization of marine by‐products.

By‐products and waste from the fish and seafood industry contain many components of key interest to be valorized, such as polyosides (chitosan), proteins (gelatins, peptides), ether extracts and fats (fatty acids, Docosahexaenoic acid, etc.), minerals and oligoelements (phosphorus, nitrogen, magnesium, calcium), pigments, etc. [71]. To date, a wide range of high value‐added products (such as chitin, chitosan, collagen, gelatin, w‐3 polyunsaturated fatty acids, enzymes, protein hydrolysates, sulfated polysaccharides, fish meal, and fish oil) have been obtained from various marine by‐products [67, 72, 73]. These bioactive molecules can be used as ingredients or additives in various industrial applications such as food, pharmaceuticals, and cosmetics [74]. Among these, many could be envisaged as useful for active packaging material developments [39, 40]. Indeed, chitosan, obtained from crustacean chitin deacetylation, and gelatin, mainly extracted from fish and cephalopod skins, as well as the fish myoproteins, have great film‐forming properties and can be cross‐linked by both physical [22, 75] or chemical [76, 77] treatments with the aim of producing biopolymers and providing structural properties to packaging films. Peptides, as well as some enzymes extracted from fish and cephalopod, have antioxidant properties that could be introduced into biopolymer matrix in order to delay oxidative degradation of film and would also migrate into food to prevent oxidation when considering active packaging films [78]. These have also antimicrobial and healing efficacies for the development of dressings and active packaging systems for the food and pharmaceutical industries [Hajji S, Ktari N, Bkhairia I, Kchaou H, Bardaa S, Chabchoub N, Boudaouara T, Boufi S, Debeaufort F and Nasri M, unpublished data].

### Active antioxidant films based on fish gelatin

The development of self‐supporting films based on gelatin or chitosan requires relative impermeability to water or aqueous solutions, including acidic solutions, in which gelatin or chitosan is highly soluble. In fact, most biopolymers, and thus the resulting films, are generally soluble in the aqueous phase, which is the main drawback. However, simply cross‐linking by chemical or physical means can compensate for this. Classically, chemical cross‐linking with aldehydes (formaldehyde, glutaraldehyde, etc.), acids (citric acid), enzymes (transglutaminases), or specific cross‐linking agents (genipin, hexamethylene diisocyanate) is possible, but the films become brittle and this leads to a loss of biodegradability and strongly limits their use in contact with food [79, 80, 81]. Cross‐linking by physical means, such as temperature and electron beam irradiation, has also been studied, but with unsatisfactory results in terms of barrier and mechanical resistance though the solubility has been significantly reduced [82].

**Fig. 2:**
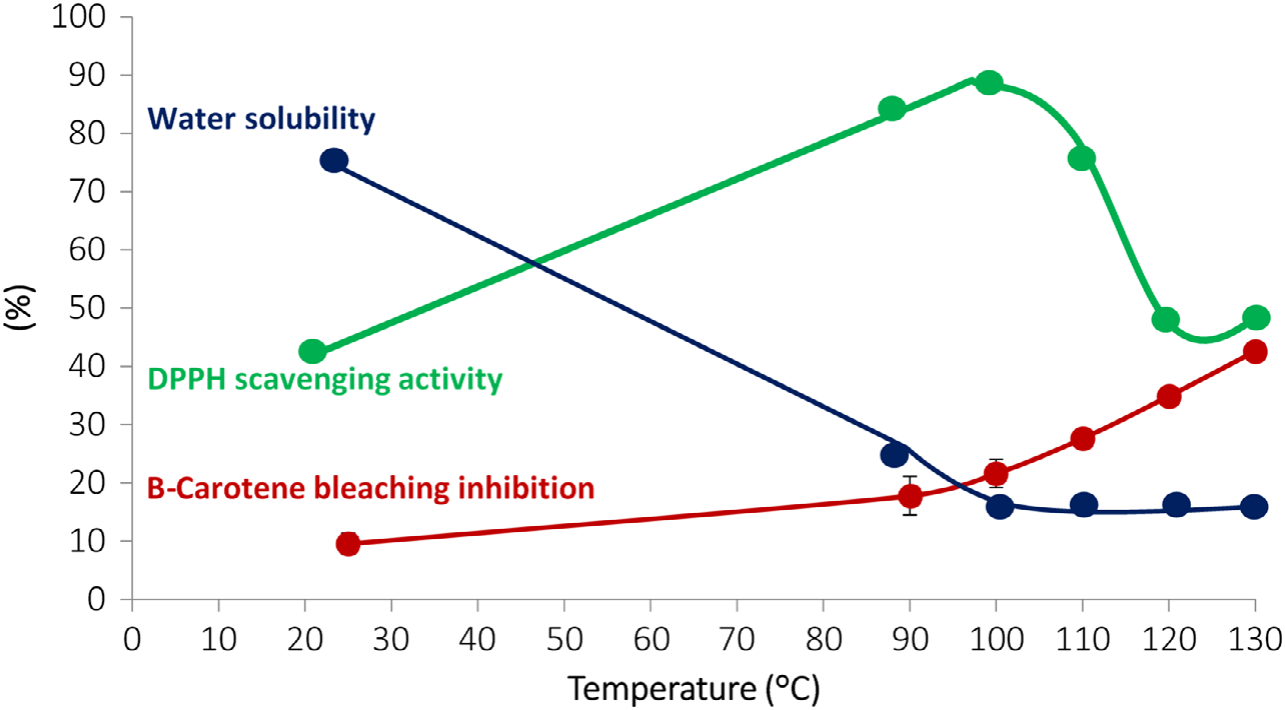
Impact of the Maillard reaction on the improvement of gelatin film water solubility and of the antioxidant properties as a function of temperature treatment (adapted from Ref. [76]).

Heat treatments in the presence of reducing sugars can be used as a complex cross‐linking process leading to the modification of protein structures and networks. This method is well known as the Maillard reaction or nonenzymatic browning reaction. The Maillard reaction is a natural and spontaneous reaction starting with the condensation of a reducing sugar with amino acids or the ԑ‐amino group of the lysyl residues of gelatin [83]. The Maillard reaction has therefore been understood as a method of both physical and chemical treatments, the main aim of which is to improve the structural and functional properties of marine gelatin‐based films [84]. Etxabide *et al*. [85] have shown that pH plays an important role in the cross‐linking efficiency of fish gelatin in the presence of lactose, and therefore, pH also plays an important role in the mechanical properties of the films. Kchaou *et al*. [76] developed films based on fish gelatin cross‐linked by the Maillard reaction at temperatures ranging from 90 to 130 °C in the presence or absence of glucose. This work thus showed a very strong reduction in the solubility of films in water, but also that the compounds resulting from the Maillard reaction had remarkable antioxidant properties [86]. These films thus exhibit either free radical scavenging properties, or metal chelation or iron reduction properties, and are therefore capable of inhibiting primary or secondary oxidation reactions depending on the temperatures and reducing sugar levels applied, as shown in Fig. 2. The films cross‐linked by the Maillard reaction have therefore been tested for the conservation of flaxseed oil (70% polyunsaturated fatty acid) in single‐dose sachets [87].

Thus, gelatin‐based films containing glucose were cross‐linked by the Maillard reaction at 120 °C and used for the design of sachets in which flaxseed oil was packaged and stored at 50 °C (accelerated tests). The results show that the peroxide value of the oil stored in the gelatin sachets was almost stable and then decreased on day 21, while the control oils showed marked oxidation, browning, and a drop in the viscosity which reveals oil hydrolysis. The application of gelatin‐based sachets appears to be an effective and interesting tool to protect oils rich in polyunsaturated fatty acids from oxidation as a biodegradable and durable package for small doses (Table 2).

**Table 2:** Flaxseed oil properties at zero time and after 21 days storage at 50 °C (accelerated tests) when stored in open vial (negative control), airtight vial (positive control) and in active gelatin films after Maillard treatment (adapted from [87]).

Property	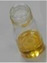 Negative control (open vial containing flaxseed oil)	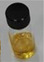 Positive control (open vial containing flaxseed oil with same headspace/oil ratio than pouches)	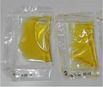 Active gelatin films heated treated (Maillard) containing flaxseed oil
Free fatty acid content (% oleic acid)			
*t* = 0 day	0.25	0.25	0.25
*t* = 21 days	1.3	0.8	0.4
Peroxide index (mm_cumene hydroperoxide eq_.per g_oil_)			
*t* = 0 day	0.4	0.4	0.4
*t* = 21 days	9.5	7.0	1.0
TBARS (mL_malonaldehyde_ per g_oil_)			
*t* = 0 day	8	8	8
*t* = 21 days	35	27	7
Absorbance at 270 nm			
*t* = 0 day	0.09	0.09	0.09
*t* = 21 days	0.45	0.30	0.16
Intrinsic viscosity estimated from NMR relaxation time (ms)			
*t* = 0 day	163	165	168
*t* = 21 days	157	162	168

### Antimicrobial films based on chitosan laminated onto plastic or biodegradable films for sliced food

**Fig. 3:**
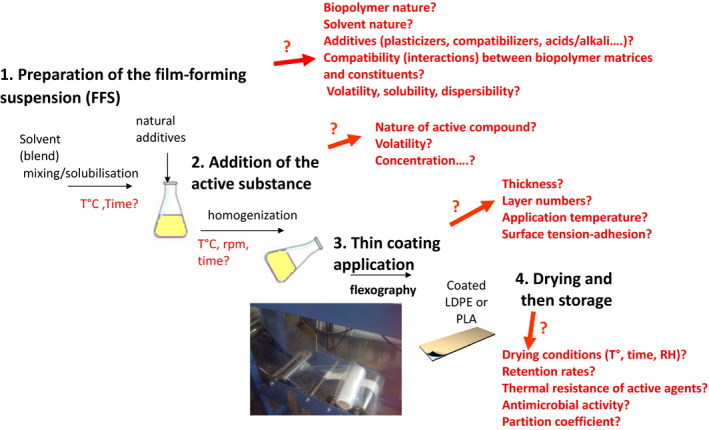
Processing steps for the development of active coating based on chitosan and essential oil components applied on industrial films (Adapted from Ref. [89]).

Chitosan and gelatin are preferred biopolymers for the manufacture of packaging thanks to their excellent film‐forming properties and their ability to be shaped by dry (extrusion ‐> films) or wet (casting ‐> coatings) processes [14, 88]. However, they have a major flaw which is their sensitivity to moisture. That said, what can be a disadvantage in terms of water vapor barrier or water solubility can be a real advantage for active packaging development. For example, a thin chitosan‐based layer applied to the inner surface (face in contact to the food) of a package will swell when exposed to the moisture of the food product and allow the release of an active molecule such as an antimicrobial. Kurek *et al*. [89, 90] developed the concept of applying a chitosan‐based coating layer containing the main compound of oregano (carvacrol) or thyme (thymol), which has antimicrobial properties, onto monopolymer films (polyethylene, polypropylene, and polyethylene terephthalate). These plastic materials can be recycled and the coating layer easily separated from the plastic support by washing with hot water, and its natural purification performed in a sewage treatment plant or by crop landfill spreading. The composition parameters, the structure of the chitosan coating layer, and its film‐forming and adhesion performances in relation to the targeted functional properties have been characterized. The importance of the design of the chitosan film and its adequacy with its support is paramount [91, 92]. The drying procedure, the optimization of formulations, and the industrial process conditions of bio‐based chitosan films are carried out as summarized in Fig. 3.

**Fig. 4:**
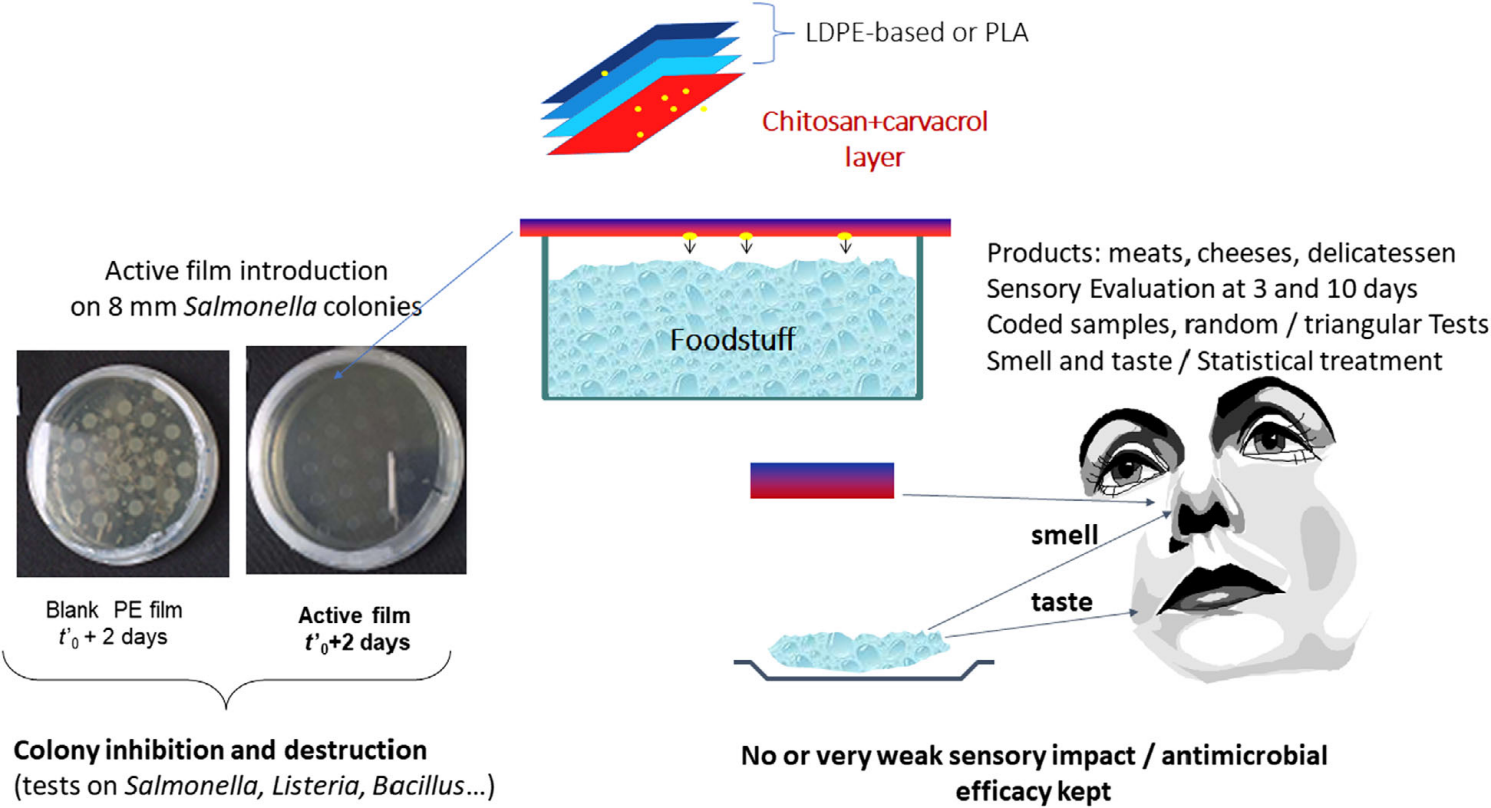
Active coated film efficacy on microbial growth and lethality and sensory impact on packaged foods (adapted from Ref. [89])

In the life cycle of an active packaging film containing volatile bioactive compounds (essential oils), there are two main stages during which the active compound can be lost: primarily, the drying stage and the storage period before application and secondarily during the use of the film (packaging). The rate of drying and relative humidity conditions of the drying air affect the structure of the chitosan network and therefore its capacity to retain the volatile active molecules. Once the film had been manufactured, it was found that at a water content of < 10% (< 50% RH), the transfers (evapo‐release of carvacrol) were slowed down sufficiently so that losses were negligible over the storage period of the active film reels, whereas at relative humidity above 75% (moisture generated by the food), carvacrol is released very quickly allowing an almost immediate antimicrobial efficacy (a few hours) when the film is applied in contact with the food or with the headspace above the food [93]. Although water slightly decreases the gas and water vapor barrier performance of chitosan‐coated films and alters the integrity of the chitosan matrix, this is desirable for the antimicrobial performance of the film. Indeed, during application, water (moisture) plays an important role in the release of the active compound from the chitosan matrix. The swelling of chitosan on contact with water vapor increases the gap between the macromolecular chains of chitosan (plasticization phenomenon) and thus facilitates the release of carvacrol. The diffusion coefficients of carvacrol in the chitosan matrix increased up to 1000 times when humidity increased from 0% to 100%. Water vapor triggered the release of carvacrol into the headspace phase. It is therefore important to control the environmental conditions when storing the active film [94]. The antimicrobial effectiveness of the films varied according to the microorganisms considered (Gram‐positive and Gram‐negative bacteria, molds, fungi) and the characteristics (composition/structure) of the chitosan layer encapsulating the carvacrol. The antimicrobial effect is controlled by the partition coefficient between the film and the headspace in the packaging [90, 94]. When the concentration released in the headspace reaches the MIC (minimum inhibitory concentration) or MLC (minimum lethal concentration), these authors found a significant efficacy (at least 6–9 log reduction) on most of the pathogenic model strains (*B. subtilis, E. coli, Listeria innocua,* and *Salmonella enteritidis*) without having a significant impact on the sensory properties of the products (cheese, meat, sliced sausage products), except on two foods that are cooked (ham and feta) [89]. Figure 4 highlights the relationship between the partition of active compounds between film and headspace, the antimicrobial efficacy, and the sensory analysis. This example shows the feasibility of active packaging based on chitosan extracted from shrimp industry waste and plant extracts. Other work is in progress on the same theme but on biodegradable packaging such as PLA using other biopolymers extracted from sea industry waste. For example, active PLA films coated with gelatin containing epigallocatechin gallate have recently been used to package fried salmon skin. This active packaging has been shown to be effective in delaying oxidation and therefore prolonging the shelf life of fried fish pieces [95].

## Conclusion: Active packaging as a potential solution for the near and far future

Valuing waste for active and sustainable packaging applications seems realistic. Numerous published works show its feasibility, especially at the laboratory scale with, for example, more than 4700 publications on the possibilities of using gelatin and/or chitosan for packaging purposes.

What is most easily achievable to date is the use of active components and biopolymers simply extracted as coatings on packaging films already industrially produced, either biodegradable films (PLA), or easily recyclable monolayer or monomaterial films (PET, polyolefins). The issues are therefore enormous, the potential even more so, but it is a path blocked by many hurdles to success: the collection of recyclable waste must be set up, extraction processes at industrial scale (scale up) must be developed, the costs must be controlled to be competitive with existing packaging systems, the implementation of ‘clean’ conversion processes (green chemistry or biorefinery) is still in its infancy, the control of health risks throughout the value chain must be controlled, the necessary adaptation of regulations is required, and consultation between scientists, producers, clients, and governments should be increased. However, one of the biggest challenges is probably the synthesis of scientific knowledge. Indeed, the scientific literature on these questions is becoming plethoric, and it is no longer possible today for a scientist to extract key or generalizable information on studied systems, extracted or tested molecules, or processes and targeted applications. The use of data compilation and processing tools, such as artificial intelligence, must be considered with the help of databases. Finally, the consumer and the citizen must be associated. Indeed, the consumer is today more and more distrustful of science and scientists as they are highly influenced by fake‐scientific news. We therefore need to educate and train future consumers, and therefore citizens, to better understand these issues and to accept future solutions, even if they are less effective but more sustainable.

So, yes: biosourced and biodegradable packaging with bioactivities that are safe for consumers’ health and for the environment and allow us to extend the shelf life of food, is the future, near on the basis of current knowledge, but still far in terms of the barriers to be overcome.

## Conflict of interest

The authors declare no conflict of interest.

## Author contributions

The manuscript, figures, and tables were conceived, written, and prepared by Frédéric Debeaufort.
